# Rituximab as a Steroid‐Sparing Therapy in Pediatric Idiopathic Pulmonary Hemosiderosis: A Case Report

**DOI:** 10.1155/crpu/3383284

**Published:** 2026-06-08

**Authors:** Abdulrahman Aldaithan, Mohamed Shehab, Mohsen Alajmi, Abdulla Alfraij

**Affiliations:** ^1^ Pediatric Intensive Care Unit, Pediatrics Division, Ahmadi Hospital, Kuwait Oil Company (KOC), Al Ahmadi, Kuwait; ^2^ Pediatric Respirology, Pediatrics Division, Ahmadi Hospital, Kuwait Oil Company (KOC), Al Ahmadi, Kuwait

**Keywords:** idiopathic pulmonary hemosiderosis, immunotherapy, IPH, pediatric, rituximab

## Abstract

**Objective:**

Idiopathic pulmonary hemosiderosis (IPH) is a rare pediatric disorder characterized by recurrent alveolar hemorrhage leading to iron‐deficiency anemia and diffuse pulmonary infiltrates. Its diagnosis is frequently delayed because early manifestations often mimic common respiratory or hematologic conditions. High‐dose corticosteroids remain the mainstay of acute management, yet prolonged steroid dependence is associated with significant sequelae. Emerging evidence suggests that B cell–directed therapy, including rituximab, may offer a steroid‐sparing alternative in refractory pediatric IPH.

**Clinical Presentation and Intervention:**

We describe an uncommon case of steroid‐dependent IPH. Her clinical course was marked by recurrent hypoxemic episodes, diffuse alveolar bleeding, and severe anemia, leading to multiple admissions to intensive care and repeated transfusion requirements. Although she received prolonged therapy with corticosteroids, hydroxychloroquine, and azathioprine, she consistently relapsed whenever steroid doses were reduced. Rituximab was introduced as a steroid‐sparing strategy, after which she demonstrated significant clinical improvement and durable remission.

**Conclusion:**

This case illustrates the diagnostic challenges of pediatric IPH when early features are incomplete and underscores the potential value of Rituximab as a steroid‐sparing option in refractory disease. The patient′s sustained remission following B cell–directed therapy supports its emerging role in IPH, though larger, well‐designed studies are needed to better define its long‐term safety and optimal use in children.

## 1. Introduction

Pediatric idiopathic pulmonary hemosiderosis (IPH) is a rare condition whose precise incidence remains uncertain; available data suggest a range of approximately 0.24–1.26 cases per million individuals per year [1]. Although IPH can occur in both adults and children, the pediatric population predominates, with a mean age of presentation of around 6 years [2, 3]. IPH should be suspected in children with recurrent or unexplained iron‐deficiency anemia, hemoptysis, and diffuse pulmonary infiltrates on chest imaging, even if all features are not present simultaneously. The disease often mimics more common conditions such as pneumonia or autoimmune disorders, leading to delayed recognition. Persistent microcytic anemia despite iron therapy, particularly in the presence of chronic cough, dyspnea, or intermittent hypoxemia together with bilateral infiltrates or ground‐glass changes on imaging and the presence of hemosiderin‐laden macrophages on bronchoalveolar lavage, strongly supports the diagnosis. [1, 4]. Histopathological confirmation via lung biopsy remains the gold standard, demonstrating siderophages (hemosiderin‐laden macrophages) in the absence of capillaritis or immunoglobulin deposition [2, 5]. In certain cases, disease progression can result in pulmonary fibrosis and ultimately culminate in end‐stage lung disease.

Management remains challenging due to frequent relapses, corticosteroid dependence, and the lack of standardized treatment protocols [6]. It primarily relies on systemic corticosteroids to control acute alveolar hemorrhage and prevent relapse, whereas immunosuppressive agents such as azathioprine, hydroxychloroquine, or cyclophosphamide are used in steroid‐dependent or refractory cases. Recent reports have highlighted the potential role of B cell–depleting therapy with rituximab as an effective steroid‐sparing option, achieving sustained remission in children with recurrent or steroid‐resistant IPH by targeting the presumed autoimmune component of the disease. Here, we report a case of steroid‐dependent pediatric IPH that achieved sustained remission following rituximab therapy.

## 2. Case Report

A previously healthy 7‐year‐old Kuwaiti girl re‐presented to our institution after two prior admissions within 3 weeks for respiratory distress and severe anemia, first documented in January 2020. There was no prior medical history or family history of similar hematologic or respiratory illness. Her initial hospitalization on January 10, 2020, was prompted by respiratory distress diagnosed as pneumonia caused by Influenza A, accompanied by profound anemia with a hemoglobin level of 5.4 g/dL. She received a single packed red blood cell (PRBC) transfusion and intravenous antibiotic therapy, resulting in gradual clinical improvement. Upon discharge, she was prescribed oral iron supplementation, with her hemoglobin level improved to 8.6 g/dL posttransfusion.

During a hematology clinic follow‐up 1 month later given her diagnosis with severe iron deficiency anemia, the patient appeared markedly pale, and testing revealed recurrent severe anemia (hemoglobin 4.6 g/dL). She was readmitted and transfused with PRBC twice, improving her hemoglobin to 10 g/dL. In this admission, she experienced intermittent oxygen desaturation requiring supplemental oxygen. A high‐resolution CT showed bilateral ground‐glass opacities with interlobular septal thickening (“crazy‐paving” pattern) that support the differential diagnosis of IPH, and pulmonary artery dilation suggestive of pulmonary hypertension (Figure 1). Bronchoscopy with BAL on February 20, 2020 revealed hemosiderin‐laden macrophages (~85%), confirming diffuse alveolar hemorrhage and establishing the diagnosis of IPH (Table 1). The patient experienced recurrent severe anemia (hemoglobin 2.8–5.4 g/dL), requiring a total of eight PRBC transfusions during disease exacerbations between January 2020 and March 2022. A summary of therapeutic regimens and transfusion parameters is provided in (Table 2). Iron studies showed low serum iron (3.4–5.5 *μ*mol/L) with markedly reduced transferrin saturation (as low as 4%), consistent with iron‐deficiency physiology in the setting of ongoing blood loss. Ferritin levels were variable (approximately 32.9–340 *μ*g/L), with higher values observed during active episodes, likely reflecting an acute‐phase response.

**Figure 1:**
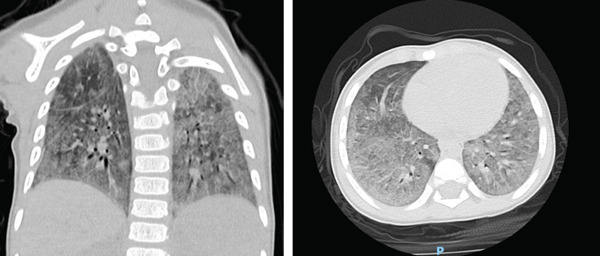
High‐resolution CT demonstrates extensive bilateral ground‐glass opacities with interlobular septal thickening, producing a crazy‐paving pattern.

**Table 1:** Diagnostic pathology and bronchoalveolar lavage (BAL) findings.

Test	Result	Clinical implication
Lung biopsy	Diffuse iron‐laden macrophages; chronic lymphoplasmacytic pleuritis; focal reactive fibrosis	Diagnostic for IPH; chronic inflammatory injury
Malignancy/infection	Absent	Supports idiopathic etiology
BAL cell distribution	95% macrophages	Hemorrhage‐driven response
BAL iron stain	**Marked hemosiderosis**	Confirms diffuse alveolar hemorrhage
Culture	No growth	Infection ruled out

*Note:* The Bold value indicates in IPH, marked hemosiderosis on BAL is evidence of chronic or recurrent diffuse alveolar hemorrhage and is one of the key pathological findings supporting the diagnosis once other causes of pulmonary bleeding have been ruled out.

**Table 2:** Summary of key therapies and transfusion parameters.

Category	Intervention	Dose and route	Duration/frequency	Key details
Corticosteroids (maintenance)	Prednisolone	10 mg PO daily (2 mg/kg/day initially)	Prolonged, with taper attempts	Steroid‐dependent course
Corticosteroids (acute)	Methylprednisolone	0.5–1 mg/kg IV q6h	5 days per episode	Used during exacerbations
Corticosteroids (pulse)	Methylprednisolone	20 mg/kg IV	3 days per pulse	Repeated during relapses (2021–2023)
Immunomodulator	Hydroxychloroquine	5–6 mg/kg PO daily	Feb 2020 – Aug 2023	Discontinued
Blood transfusion	Packed RBCs	100–300 mL IV (=10–15 mL/kg)	8 episodes (2020–2022)	During severe anemia/exacerbations
Immunosuppressant	Azathioprine	25 → 50 mg PO daily	Aug 2023–Jan 2025	Steroid‐sparing attempt
**Biologic therapy**	**Rituximab**	**375** mg**/m** ^**2**^ **(≈320–360** mg**) IV**	**~4-6** months **(maintenance)**	**Initiated May 2024; ongoing**

*Note:* Rituximab was bolded to indicate that it is the patient’s current ongoing therapy and the only treatment that has been continued. Its introduction was associated with successful withdrawal of corticosteroids and other immunosuppressive agents while maintaining disease control.

In May 2024, the patient was transferred to a facility with expertise in such a rare condition for further investigation and confirmation. Lung biopsy confirmed diffuse alveolar hemosiderosis with chronic lymphoplasmacytic pleuritis and reactive focal fibrosis, without evidence of malignancy or infectious pathology. BAL performed at that time showed macrophage‐predominant cytology (95%) with marked hemosiderosis and sterile cultures. Rituximab therapy was initiated, resulting in a favorable clinical response. A 1‐year comparison of the patient′s clinical course before and after rituximab initiation demonstrated a marked improvement in disease control. During the year prior to rituximab therapy (May 2023–May 2024), she required three hospital admissions for disease exacerbations. In contrast, throughout the subsequent year following rituximab initiation (May 2024–May 2025), she had no hospital admissions, required no pulse corticosteroid therapy, and was managed exclusively through routine outpatient follow‐up without the need for emergency interventions or rescue medications (Figure 2). Notably, the duration of systemic corticosteroid therapy prior to rituximab initiation was prolonged, with multiple attempts at tapering resulting in relapse. Despite prolonged corticosteroid exposure, height velocity remained within normal age‐adjusted ranges, without evidence of clinically significant growth suppression (Figure 3). Following rituximab therapy, corticosteroids were successfully discontinued. During follow‐up, the patient demonstrated sustained clinical and laboratory stability, with hemoglobin and iron indices remaining within acceptable ranges (e.g., hemoglobin 13 g/dL and ferritin 132 *μ*g/L on September 2025), alongside radiologic stability, consistent with a favorable response to rituximab (Figure 4).

**Figure 2:**
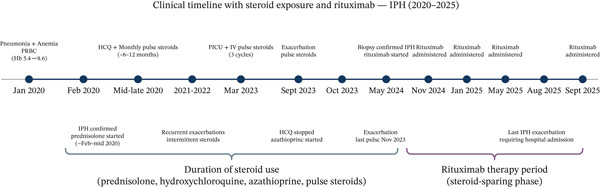
Clinical timeline of disease course and therapeutic interventions.

**Figure 3:**
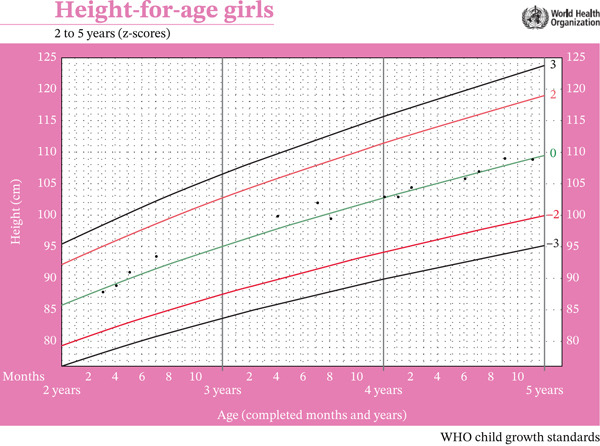
Longitudinal height‐for‐age growth chart demonstrating preserved growth trajectory during corticosteroid therapy.

**Figure 4:**
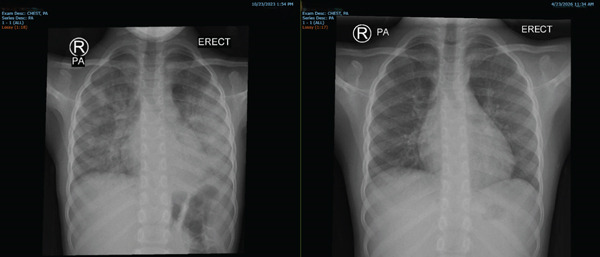
Comparative chest radiographs demonstrating interval improvement in pulmonary infiltrates following rituximab therapy (left: prior to rituximab initiation; right: during follow‐up on rituximab).

## 3. Discussion

The diagnosis of IPH in children remains challenging due to its nonspecific and often incomplete clinical presentation. Early episodes are frequently misattributed to common respiratory illnesses such as pneumonia or anemia of chronic disease, delaying recognition until the classical triad of iron‐deficiency anemia, hemoptysis, and diffuse pulmonary infiltrates becomes evident. In our case, the initial hospitalizations were interpreted as pneumonia with unexplained severe anemia, leading to delayed diagnosis until the recurrence of profound anemia and characteristic radiologic findings prompted further evaluation. Such delays are clinically significant, as missed or late recognition of IPH may result in repeated alveolar hemorrhage, progressive pulmonary fibrosis, and pulmonary hypertension, all of which can worsen long‐term outcomes. High‐dose pulse corticosteroid therapy remains the cornerstone of acute management, aiming to suppress immune‐mediated alveolar injury and halt active bleeding through rapid anti‐inflammatory action and high peak plasma concentrations. Despite its efficacy, prolonged or repeated steroid exposure is associated with well‐documented adverse effects, including growth suppression, bone demineralization, hypertension, glucose intolerance, and increased infection risk, underscoring the need for alternative or steroid‐sparing immunomodulatory agents. Furthermore, some patients could experience relapse despite therapy or during dose tapering due to adverse effects, necessitating the consideration of alternative treatment modalities [7].

Rituximab, an anti‐CD20 monoclonal antibody, is utilized to attenuate B cell–mediated autoimmune activity in IPH [8]. It has primarily been employed as a steroid‐sparing agent in cases where corticosteroid toxicity becomes evident, rather than as a first‐ or second‐line therapeutic option for IPH [9]. Rituximab is a well‐tolerated and effective biologic agent that has been widely utilized in the management of various autoimmune disorders [10]. It may be effective in IPH because B cell depletion reduces the underlying immune dysregulation reflected by nonpathogenic autoantibodies, thereby dampening the aberrant lymphocyte‐driven inflammatory cascade that leads to alveolar hemorrhage [11]. Recurrent relapses with alveolar hemorrhage and oxidative stress from unbound free iron can ultimately lead to pulmonary fibrosis, so early initiation of rituximab may help mitigate this process and delay fibrotic progression [12]. Despite this rationale, the rarity of pediatric IPH limits the feasibility of adequately powered prospective studies; however, this report may help inform and stimulate future well‐designed multicenter investigations evaluating therapies such as rituximab.

## 4. Conclusion

This case underscores the therapeutic potential of rituximab in managing refractory IPH in children, achieving sustained remission and minimizing corticosteroid dependence in a patient unresponsive to conventional immunosuppressive therapy. Early integration of rituximab into the treatment algorithm for severe or steroid‐dependent IPH may offer improved long‐term outcomes and a better quality of life for affected patients. Given the rarity of pediatric IPH, this report may help guide and stimulate future well‐designed multicenter studies evaluating rituximab.

## Author Contributions

**Abdulrahman Aldaithan:** conceptualization, methodology, data curation, writing—original draft, and supervision; **Mohamed Shehab:** writing—original draft; **Mohsen Alajmi:** conceptualization, writing—review; **Abdulla Alfraij:** methodology, writing—original draft, review.

## Funding

No funding was received for this manuscript.

## Ethics Statement

Written informed consent was obtained from the father (the guardian) for the publication of his child′s condition as a case report.

## Consent

Written informed consent was obtained from the guardian (the father) for publication of this case report and any accompanying images. A copy of the written consent is available for review by the editor in chief of this journal.

## Conflicts of Interest

The authors declare no conflicts of interest.

## Data Availability

The data that support the findings of this study are available from the corresponding author upon reasonable request.
